# Styptic Cotton

**Published:** 1872-09

**Authors:** 


Styptic Cotton.—Dr. Rohland, of New-York (*The Medical Record*) has prepared a styptic cotton (*Gossypium Stypticum*) that promises to be a useful addition to the means of arresting passive hemorrhages from extensive surfaces. It has the advantage of cleanliness and convenience, and possesses no irritating properties. It is prepared by boiling the cotton in a solution of alum and gum of benzoin, and, after the cotton is dried and picked, it is saturated by perchloride of iron. It is put up in boxes of convenient size.

